# Comparing the impact of recess duration on motor coordination and psychological variables: An exploratory study

**DOI:** 10.1371/journal.pone.0361479

**Published:** 2026-11-04

**Authors:** Hailey G. von Borck, Phillip Esposito, Robyn Braun-Trocchio, Lauren M. Wagner, Deborah J. Rhea

**Affiliations:** Department of Kinesiology, Texas Christian University, Fort Worth, Texas, United States of America; Southwest University, CHINA

## Abstract

Recess serves as a productive environment for children to play independently of adults, which promotes physiological and psychological development. However, current recess durations in schools across the United States (U.S.) are frequently insufficient in providing opportunities for physical activity (PA) to promote developmental improvements. One of the most well-studied evidence-based recess interventions, the LiiNK Project^®^, has demonstrated that when children receive 60 minutes of recess daily, motor coordination (MC) is significantly better than when children receive 30 minutes daily. However, child outcomes are lacking when schools modify the recess intervention duration in older grades to 30 minutes daily while continuing to practice the protocols and procedures from the full intervention. Therefore, the primary purpose of this study was to explore observed physiological (MC) and psychological (self-esteem and perceived physical ability) differences and associations between fourth and fifth grade children who received a modified version of the LiiNK intervention (*n* = 120) and children who did not receive the modified intervention (*n* = 98) at two-time points in one school year. The Körperkoordinationstest für Kinder (KTK) was used to assess MC, while the KID-KINDL self-esteem subscale and the Perceived Physical Ability Scale (PPASC) were used to assess the psychological variables. Grade, sex, race, and body fat percentages were collected to describe any group differences for a better interpretation of the groups. A mixed two-way repeated measures MANOVA analysis identified a between-group PPASC difference (p < 0.001), while the control group reported higher scores at both time points, suggesting a pre-existing group difference rather than a modified intervention change. Further, significant within-subjects time-related improvements were observed for single-leg hop (SH) and sideways stepping (SS), while a significant time-related decline was observed for self-esteem. Lastly, 2 x 2 Pearson chi-square analysis of “Normal-to-Great” and “Below Normal” MQ classifications was conducted separately at each time point. Although there were no significant findings, descriptively, both groups at Time 1 (85.8% of the modified intervention group and 90.8% of control group) were classified as “Normal-to-Great” MQ, improving to 88.3% and 95.9%, respectively, at Time 2. The “Below Normal” MQ classification decreased from 14.2% to 11.7% and 9.2% to 4.1% in the two groups respectively. Overall, these results provide preliminary insights into observed patterns of physiological and psychological outcomes for fourth and fifth grade children with similar recess minutes, but different protocols daily. However, these results provide no evidence that the modified intervention benefits the children any better than having 30 minutes of recess without the modified protocols. Future studies should compare the modified 30-minute intervention with a larger sample of children in the full LiiNK intervention (60 minutes) to assess these same variables, as well as collect the second data point in May instead of February.

## 1. Introduction

Physical inactivity and sedentary behaviors are notably high posing a significant health challenge. This is particularly apparent in children, whose physiological and psychological development depend on regular, active daily movement. Inactivity and sedentary behaviors are commonly associated with poor body composition, lower self-esteem, and deficient motor skill development in children [1–3]. Elementary schools, where children spend most of their day, offer the most equitable and safe setting, to promote daily outdoor breaks through recess [4]. Recess is defined as unstructured outdoor play where children are self-directed and self-controlled in a safe environment [4]. This type of play is instrumental in limiting sedentary behaviors and naturally promoting moderate-to-vigorous physical activity (MVPA) [5,6]. It also plays a crucial role in improving motor coordination (MC) opportunities, refining kinesthetic perception, and activating the child’s brain through dynamic and complex repetitive tasks such as running, jumping, and climbing [4,7–9].

Recess serves as a productive environment for children to play independently of adults, which promotes physiological and psychological development. However, current school recess durations across the United States (U.S.) are frequently insufficient to provide adequate opportunities for children to be physically active daily [5,8–10]. Evidence indicates that reduced recess is associated with deficits in movement patterns, specifically motor skills influencing children’s MC [11–13]. MC refers to the ability to execute controlled, whole-body movements involving balance, agility, gross motor skills, and postural control, and is particularly noticeable in recess settings [12–14]. Theoretical frameworks such as the Ecological Dynamics Approach (EDA) offer valuable insight into how motor behaviors emerge through the interaction of individual, environment, and task constraints [15]. EDA utilizes the concept that movement skills are not pre-determined but develop over time through adaptive interactions within diverse physical constructs. School recess is an ideal environment for these interactions, allowing children to independently choose to participate in various tasks that naturally encourage MC development [8,15–17].

In contrast, limited opportunities for recess have been associated with delays in motor development, increase the risk of coordination disorders, and negatively impact children’s confidence and willingness to engage in physically demanding activities [11,13]. Ultimately, when providing recess that is unstructured and outdoors, children have shown to develop physical skills such as MC through many diverse movement opportunities as well as boost psychological components such as perceived physical ability which can positively influence self-esteem [12,18–21]. Perceived physical ability is defined as an individual’s personal assessment of their own competence in strength, endurance, agility, and other physical tasks and activities [19]. Self-esteem is defined as an individual’s overall sense of self-worth across various life domains and is often linked to children’s motivation to participate in physical and social activities [22–26]. Farhat et al. [18] found that children with MC disorders reported diminished perceived physical abilities and lower participation in PA than those with normal-to-good MC skills. The active, social context of recess provides children with opportunities for task mastery and peer support, which are vital for shaping perceived physical ability and self-esteem [27,28]. Conversely, decreased access to recess contributes to social isolation, leading to feelings of inadequacy and decreased emotional regulation in children [28–30]. Together, these findings reinforce the importance of integrating MC (physiological) with perceived physical ability and self-esteem (psychological) when investigating children’s responses to school-based recess interventions.

Over the past few years, recess interventions of different durations have become more evident, focused on different physiological and psychological components [31]. One of the most well-studied recess interventions, the LiiNK Project^®^, involves the implementation of four 15-minute recess breaks and 15-minute character development lessons daily for each sequential academic year after three teacher and administrator trainings have taken place [5]. Previous full LiiNK intervention studies (60 minutes of recess) compared to schools with 30 minutes or less of recess have shown a 40% reduction in off-task behaviors in one year [32], while also showing significantly improved positive social behaviors and attentional focus [33,34], and a significant decline in chronic stress [35]. Additionally, the intervention children have recorded approximately 145 minutes of MVPA daily at school, more than twice the recommended 60 minutes of MVPA recommended daily for elementary school children [9,36]. Moreover, children participating in the full LiiNK intervention have reflected significantly better MC skills than children in the control group with 30 minutes or less of recess daily [16,31], while 85% of the full intervention group scored within the “Normal-to-Great” MQ range reflecting significantly higher percentages than children in the control group at 79% [13,16].

The LiiNK results assessing MC used the Körperkoordinationstest für Kinder (KTK), an originally developed and validated international MC assessment tool [37]. It was found to be a reliable instrument for classifying MC levels and identifying developmental delays internationally, followed by demonstrating its feasibility and fidelity as well as identifying developmental delays in U.S. elementary physical education (P.E.) contexts [16,38–40]. However, child outcomes are lacking when schools modify the recess intervention duration in older grades to 30 minutes daily while continuing to practice the protocols and procedures from the full intervention. Prior research has reported associations between KTK-measured MC and selected constructs such as self-esteem and perceived physical ability [41–44]. Longitudinal KTK evidence indicates that MC is a significant predictor of global self-esteem during childhood, with higher MC levels associated with better self-esteem [41] Although the association is weak, Lopes et al [41] found that MC categories within the KTK had a mitigating effect on the decrease of self-esteem, suggesting that promoting MC during childhood may positively influence children’s psychological well-being and encourage consistent participation in physical activities during recess. Finally, previous findings suggest that MC may be associated with the maintenance of self-esteem and perceived physical ability during childhood and early adolescence; however, the strength and direction of these relationships may vary across populations and developmental periods [41,45].

Given this established relationship, the recess environment represents a relevant setting for exploring relationships among recess duration, MC, and psychological outcomes. The full LiiNK intervention has shown across numerous studies that children demonstrate favorable physiological and psychological patterns when they are provided recess sessions throughout the day rather than only 30 minutes or less daily. However, some school districts, following the intervention’s completion, have elected to reduce the recess duration to 30 minutes (two 15-minute breaks daily) in fourth and fifth grade children due to factors such as testing pressures, but continued to do the character lessons and LiiNK protocols. Due to the lack of studies examining physiological and psychological components with this modified LiiNK intervention recess duration, exploration of this modification is warranted.

Therefore, the primary purpose of this study was to explore observed physiological (MC) and psychological (self-esteem and perceived physical ability) differences between fourth and fifth grade children who received a modified version of the LiiNK intervention and children who did not receive the modified intervention at two-time points in one school year (September and February). A secondary purpose was to explore potential MC proficiency to disordered ability level differences and associations between the two groups.

## 2. Method

### 2.1. Study design and participants

#### 2.1.1. Study design.

This study was a non-randomized, comparative observational design which included fourth and fifth grade children from six elementary schools across two Texas districts. Children were not randomly assigned to study conditions; rather, group membership was determined by the district and school recess practices already in place. Three schools participated in the modified version of the LiiNK Project intervention (30 minutes instead of 60 minutes of recess). The three schools representing the control group were provided one 30-minute recess. Accordingly, comparisons between groups were considered exploratory and were not designed to establish causal modified intervention effects. The Time 1 and Time 2 assessments were completed in September 2024 and February 2025.

The intervention protocols included the following components: (1) recess could not be withheld for punishment or tutoring; (2) the 15-minute recess period was strictly measured from the moment children exited the building until the conclusion of the 15 minutes on the playground, with transition time to and from the classroom excluded from this duration; (3) children were allowed to participate in outdoor recess across a “feel” temperature range (13°F to 103°F); (4) all children were allowed to go outside for recess, including those with injuries or illness, unless physician provided documentation stated otherwise; and (5) children participated in a mandatory 15-minute daily character development lesson using a year-long developmentally appropriated curriculum for each grade level called Positive Action^®^.

In contrast, the control school’s protocols were: (1) recess could be withheld for punishment and tutoring; (2) the 30-minute recess period included transition from the classroom to the playground and back which reduced the recess duration to less than 30 minutes; (3) outdoor recess was permitted only during moderate temperatures, specifically within the “feel” range of 50°F to 90°F; (4) children who were sick or injured were not allowed to participate in recess activities, regardless of their preference; and (5) a character development curriculum was not used at all.

The inclusion criteria for this study were: (1) parental consent and child assent were obtained before Time 1 testing; (2) children were required to be in fourth or fifth grade (ages nine to eleven years); (3) children needed to be fluent in both reading and speaking English; (4) children had to exhibit full body control and have no present injuries that would stop them from participating during the P.E. period; (5) children were required to complete the questionnaire within the designated P.E. period time frame; (6) children needed to be present for Time 1 and Time 2 to complete the four physical assessments and computer-based psychological instruments.

#### 2.1.2. Participants.

A total of 218 children in fourth and fifth grade across District 1 (*n* = 120) and District 2 (*n* = 98) participated in this exploratory study during the 2024–2025 school year. The populations were relatively similar between the modified intervention group (District 1) and the control group (District 2). Since children were not randomly assigned to different conditions, demographic characteristics were collected to describe any potential between-group differences that could influence the interpretation of the exploratory findings.

Table 1 reflects the demographics for each group by sex, grade, race, and body fat percentage. One notable group variation showed the modified intervention group had a higher percentage of Hispanic children (44%) while the control group had a higher percentage of White children (67%).

**Table 1:** Participant demographics for group, grade, sex, race, and time 1 test and time 2 test body fat percentage.

Variable		Intervention *(N)*	Control *(N)*	Total *(N)*
Sex	Female	65	49	114
	Male	55	49	104
	**Total**	**120**	**98**	**218**
Grade	Fourth	52	53	105
	Fifth	68	45	113
	**Total**	**120**	**98**	**218**
Race	White	36	66	102
	Hispanic	53	28	81
	Black or African American	18	4	22
	Asian	11	0	11
	American Indian/Alaskan Native	2	0	2
	**Total**	**120**	**98**	**218**
Time 1 BF%	Healthy	66	68	134
	Overfat	18	14	32
	Obese	36	16	52
	**Total**	**120**	**98**	**218**
Time 2 BF%	Healthy	69	65	134
	Overfat	17	19	36
	Obese	34	14	48
	**Total**	**120**	**98**	**218**

Note. BF%: Body Fat Percentage.

Body fat classification differences were also reflected in Table 1. Specifically, the modified intervention group showed a 2.5% increase of children classified as healthy from Time 1 to Time 2, while a 0.8% decrease in the overfat category and a 1.7% decrease was observed in the obese category were observed. In contrast, the control group demonstrated a 3.1% decrease in the healthy classification from Time 1 to Time 2, while a 5.1% increase was observed in the overfat category, and a 2.0% decrease was observed in the obese category. These values were used to describe the sample and were not interpreted as effects of the recess condition because body fat classifications were not included as adjusted covariates in the preliminary analyses.

An a priori power analysis was conducted using G*Power 3.1 using the F-test family and the repeated-measures ANOVA, within–between interaction procedure. The analysis reflected a design with two groups and two measurement occasions and specified an assumed effect size of *f* = .06, *α* = .05, and statistical power of *1 − β* = .80. Since only two repeated measurements were included, the nonsphericity correction was ε = 1.00. The correlation among repeated measurements entered in the original G*Power analysis was not retained and therefore cannot be reported with certainty. The original sample consisted of 263 children, with anticipated attrition between the two data collection periods. Attrition accounted for 17.1% of the children due to illness, withdrawal from the study, or relocation, which reduced the final sample size to 218 children.

### 2.2. Instruments

#### 2.2.1. Körperkoordinationstest für Kinder (KTK).

The KTK is designed to assess the general MC variables of agility, speed, postural control, balance, strength, rhythm, and laterality through four subtests: walking backward (WB), single leg hop (SH), lateral jump (LJ), and sideways stepping (SS) [30,45]. The KTK has demonstrated excellent test-retest reliability for the total raw score (*α* = 0.95) [16]. The KTK subtest scores have also noted consistent reliability across several countries, WB*: α* = 0.88*,* SH*: α* = 0.93*,* SS*: α* = 0.96*,* and LJ*: α =* 0.94.

The standardized scoring of this assessment tool is to generate motor quotient scores (MQ), offering a reliable and valid metric for evaluating MC relative to age-specific developmental benchmarks. These scores are developed from the four subtest raw scores to assess severe MC disorders to great MC abilities. Following the calculation of raw scores from the four subtests, each score is converted into an individual corresponding MQ score using standardized conversion tables originally developed in Germany and subsequently translated and validated through a previous U.S. based feasibility study [16,30]. The four individual MQ scores were summed to generate a total MQ score, normalized by the child’s age and sex-based normative data. Once the total MQ score was established, a child’s motor coordination ability level, ranging from severe gross motor coordination disorder to great gross motor coordination, was determined. For descriptive reporting, all five Gesamt-MQ classifications (Great, Good, Normal, Moderate Disorder, and Severe Disorder) were retained. For the secondary chi-square exploratory analysis, classifications were grouped as “Normal-to-Great” (Normal, Good, and Great) and “Below-Normal” (Moderate and Severe).

**Subtest 1: Walking Backward (WB):** This subtest measures vertical balance, rhythm, and strength by having children walk backward barefoot on three balance beams of decreasing width (6.0 cm, 4.5 cm, 3.0 cm; each 7 ft long) [16,30]. Starting from a wooden platform, children have three attempts per beam, with up to eight heel-to-toe steps per attempt (excluding the initial plantar step), for a maximum score of 72. The test takes approximately three minutes per child. Reliability from Time 1-test to Time 2-test used Cronbach’s α = 0.86 for this study.

**Subtest 2: Single Leg Hop (SH):** This subtest assesses vertical strength, rhythm, and balance by having children hop over foam pads of increasing height (in 5 cm increments) using each leg independently [16,30]. Up to 12 pads (60 cm max height; each 60 x 20 x 5 cm) are stacked on a tri-fold gym mat for stability. The starting heights for this study were 10 cm for fourth graders and 15 cm for fifth graders. Children had three attempts per leg at each height, earning 3, 2, or 1 point based on the attempt in which they succeeded; no success after three attempts ended further trials for that leg. A maximum of 36 points per leg (72 total) was possible. The test took approximately five minutes per child. Reliability from Time 1 and Time 2 used Cronbach’s α = 0.90.

**Subtest 3: Lateral Jump (LJ):** This subtest assesses a child’s lateral speed, rhythm, and agility by measuring the ability to perform synchronized and stable side-to-side jumps over a wooden obstacle. The lateral jump is evaluated using a wooden obstacle (5 ft 10 in long by 24 in wide) [16,30]. The task was performed twice within a 15-second interval, using a smartphone application timer. If the child stepped on the wooden divider or jumped with both feet not crossing the wooden divider, the jump would not be applied to the final score. The total score was calculated by summing the scores from both attempts. Reliability from Time 1 to Time 2 used Cronbach’s α = 0.85.

**Subtest 4: Sideways Stepping (SS):** This subtest evaluates lateral speed, rhythm, strength, and balance during the sequential movement which determines the child’s capacity to execute the subtest efficiently while maintaining postural stability. The task requires two wooden platforms (25 cm x 25 cm x 5.7 cm; L x W x H). The task involves repeatedly lifting one of the wooden platforms and placing it adjacently then stepping on that platform to create a walking path [16,30]. This subtest was repeated twice, with each attempt lasting 20 seconds with an additional 10 second optional break between the two trials using a smartphone application for the timer. Points were awarded for correctly using both hands to move and place the platform, as well as for stepping onto the newly placed platform with both feet. The total score was calculated by adding the scores of both attempts. Reliability from Time 1 to Time 2 used Cronbach’s *α* = 0.82.

#### 2.2.2. Bioelectrical Impedance Analysis (BIA).

The BIA scale used in this study was the TANITA BF 2000, which demonstrated consistency in measuring body composition and body fat in children [2,46]. Kabiri et al. [46] reported high-test retest reliability for BIA body fat percentage readings with an intraclass correlation coefficient *ICC* = 0.999 (95% *CI* = 0.999, 0.999). For this study, body fat percentage was assessed at both time-points to describe the sample, classifying children into four categories: obese, overfat, healthy, and underfat. The classifications “overfat” and “underfat” correspond to the commonly recognized terms “overweight” and “underweight,” respectively. This is due to BIA assessing fat and not weight in children. Additionally, previous research combined the “underfat” and “healthy” classifications of children into the healthy body fat classification, while keeping overfat and obese as individual classifications [2,47]. Therefore, these three classifications were reported in this study as well. The BIA scores from Time 1 to Time 2 reflected Cronbach’s value of 0.93 indicating that the scores were highly reliable.

#### 2.2.3. Psychological assessments.

**KID-KINDL Self-Esteem Subscale:** The KID-KINDL is a widely used self-reporting instrument designed to assess the health-related quality of life of children and adolescents aged eight to sixteen years [48]. For this study, the self-esteem subscale was utilized as it can be assessed independently from other scales in the KID-KINDL. This subscale has typically demonstrated a moderate reliability, Cronbach’s *α* = 0.75, in other studies and convergent validity has been supported by moderate to strong positive correlations (*r* = 0.71–0.83) [48]. This subscale consisted of four items which took less than five minutes to complete. The four questions were assessed on a 5-point Likert scale ranging from 1 (never) to 5 (all of the time), with no items reversed. The subscale total score ranged from 4–20. In this current study, self-esteem was found to be Cronbach’s *α* = 0.66 which is considered marginally reliable and lower than other studies with similar age groups.

**Perceived Physical Ability Scale for Children (PPASC):** The PPASC assessed children’s perception of their physical ability such as speed, strength, and agility [19]. The scale was revised to six items to specifically identify strength, speed, and coordinative abilities in children [19]. These six items were assessed on a 4-point Likert scale ranging from 1 (I run very slowly) to 4 (I run very fast). Higher PPASC scores indicated a greater self-perception of physical ability, while lower scores reflected a lower self-perception. The PPASC has shown to have good reliability in children with a Cronbach’s *α* = 0.84 [19]. The PPASC has also shown to be a valid tool with strong external and internal validity assessed in male and female children (*r* = 0.72) [19]. The PPASC in this study was comparable to the other studies with a Cronbach’s *α* = 0.80.

### 2.3. Procedures

The University Institutional Review Board (IRB) approved the current study (1801-065-1801), followed by each school principal and physical educator consent. A school administrator from each school provided information on biological sex, grade level, and race prior to collection.

#### 2.3.1. Time 1.

Following approval, three LiiNK researchers were trained to administer the physiological and psychological assessments. Children were eligible to participate if parental consent, child assent, and the inclusion criteria were met. During Time 1, the primary researcher and the LiiNK research team arrived 30 minutes in advance to set up four stations: one for LJ and SS, and one for BIA and questionnaire administration, one for WB, and the last for SH.

Prior to the children arriving, the LiiNK research team were assigned to one of three stations: one researcher to Station 1 (LJ and SS), one researcher to Station 2 (BIA and questionnaire), and one researcher to Station 3 (WB). Once assessments were completed at Stations 1 and 3, each of the researchers from Stations 1 and 3 moved to Station 4 (SH) to complete this assessment.

Once the children arrived at their assigned spots on the floor for class, one LiiNK researcher explained the objectives of each physical and psychological task and obtained child assent from each child prior to assigning the children to one of the three stations to begin the rotation. The children moved to Station 4 as they completed Station 1 or 3. Upon completion of the four assessment stations, researchers verified all data had been collected and confirmed that each child had completed the Time 1 testing in its entirety. If a child had not completed all stations, the primary researcher would make sure the stations were completed on make-up days if the children were in attendance.

#### 2.3.2. Time 2.

Before the Time 2 testing, two additional LiiNK researchers received training on the administration of the physiological and psychological assessments, while the original three researchers underwent follow-up training. Consistent with Time 1 procedures, the primary researcher and the LiiNK research team arrived 30 minutes early to set up the four assessment stations.

Upon arrival and leading up to beginning the station assessments, the same LiiNK researcher from Time 1 reiterated the objectives and instructions for each station. The same testing protocol and station procedures used at Time 1 were used at Time 2. Due to the increased number of researchers from Time 1 to Time 2, the stations were set up and the number of researchers per station were adjusted. Two researchers were assigned to Stations 1 (LJ and SS) and 2 (BIA and questionnaire), and one researcher to Station 3 (WB). Once assessments were completed at Stations 1 and 3, the same procedures were followed for testing at Station 4, verifying all data had been collected, and setting extra time for assessment completion if needed.

### 2.4. Data analysis

Data was analyzed using the IBM Statistical Package for Social Sciences (SPSS) Version 29. Descriptives were reported for grade, sex, race, and body fat to describe the sample. Means and standard deviations were calculated for the dependent variables (KTK subtests, KID KINDL self-esteem (SES) subscale, and PPASC), to describe the sample by group (modified intervention and control) for Time 1 and Time 2.

Prior to the primary analyses, dependent-variable distributions were examined at the univariate level, and assumptions relevant to the repeated-measures MANOVA were evaluated, including homogeneity of covariance matrices, homogeneity of error variances, and potential multicollinearity among the dependent variables. Univariate outliers were screened within groups using standardized scores, with |*z*| > 3.29 used as a conservative flag for further inspection. Since formal multivariate normality testing was limited, normality was considered cautiously using available univariate diagnostics. As well, covariance homogeneity was not supported, so Pillai’s Trace was used as the primary multivariate statistic because of its greater robustness to this assumption violation. Due to only two time points, sphericity was inherently satisfied.

Reliability was assessed using Cronbach’s alpha for MC subtests, the self-esteem subscale and the PPASC at each time point. All statistical analyses were conducted using a significance level of *p* < 0.05. Omnibus multivariate effects were interpreted before outcome-specific follow up tests. When outcome specific follow up tests were warranted, a Bonferroni-adjusted criterion α = .0083 was used across six dependent variables, while *p* < 0.05 was retained for omnibus and other exploratory analyses. Effect sizes were calculated using partial eta squared (*η²*_*p*_) and Cohen’s d to assess the magnitude of observed differences. Ninety-five percent confidence intervals for partial eta squared (*η²*_*p*_) were calculated from the corresponding F statistics using the non-central F distribution.

To explore the primary purpose examining physiological (MC) and psychological (self-esteem and perceived physical ability) differences between the two groups across two time points, a mixed two-way repeated measures MANOVA was conducted to examine the group and time differences for MC (four KTK subtests) and psychological factors (self-esteem and perceived physical ability). This approach allowed simultaneous examination of six related outcomes for potential group, time, and group x time effects while reducing Type 1 error compared with conducting separate analyses for each outcome. The analysis explored multivariate and univariate differences within-subjects effects (comparing Time 1 to Time 2 scores within every child) and between-subjects effects (comparing the modified intervention and control groups).

Additionally, for the second exploratory purpose, Pearson chi-square tests were conducted separately in sequential order at Time 1 and Time 2 to compare the “Normal-to-Great” (normal, good, and great gross motor coordination) and “Below Normal” (moderate to severe gross motor coordination disorder) MQ classification groups. To avoid pooling repeated observations from the same children, each time point was analyzed as a separate 2 x 2 contingency table so that each child only contributed to one observation. Since two-time-specific chi-square tests were conducted, a Bonferroni-adjusted significance criterion of *α* = 0.025 was used. These analyses were intended to explore descriptive between-group differences in motor-coordination classifications at each time point rather than to test within-child over time. All statistically significant findings were interpreted with emphasis placed on the magnitude of effects, trends, and the descriptive associations and differences observed.

## 3. Results

### 3.1. Descriptive and exploratory group differences

Preliminary assumption normality screening was conducted before the two-way mixed repeated measures MANOVA. Univariate normality was assessed using Shapiro-Wilk tests which found significance for WB (*p* < 0.001), self-esteem (*p* < 0.001), and perceived physical ability (Time 1: *p* < 0.009; Time 2: *p* < 0.001) at both Time 1 and Time 2 (*p* < 0.05). Whereas SH, LJ, and SS did not demonstrate significant departures from normality. Across all dependent variables and time points, skewness and kurtosis values were relatively modest across all dependent variables, and the corresponding means, trimmed means, and medians were generally similar. Univariate outliers were screened within each group using standardized scores, with **|***z*| **>** 3.29 used as a conservative flag for further inspection. Given the relatively large sample (N = 218), the sensitivity of the formal normality tests to small distributional deviations, and the exploratory nature of the study, these departures were not considered significant to preclude the two-way mixed repeated measures MANOVA. Box’s M was significant, *M* = 130.30, *F*(78, 135766.46) = 1.57, *p* < 0.05), indicating that equality of covariance matrices could not be assumed. Therefore, Pillai’s Trace was used as the primary multivariate statistic because of its relative robustness to violations of covariance of homogeneity.

The MANOVA revealed significant between-subjects group differences, Pillai’s Trace *V* = 0.09*, F* (6, 208) = 3.57*, p* < 0.01*, η²*_*p*_ *=* 0.09, 95% CI [0.01, 0.15] with about 9.2% of the variance explained for the physiological and psychological variables used. Table 2 reflects Time 1 and Time 2 group descriptive differences for the KTK subtests, KID-KINDL subscale (self-esteem), and PPASC. Given multiple comparisons and to minimize making a Type I error a Bonferroni-adjustment criteria of the six outcome-specific follow up tests, perceived physical ability was the only variable demonstrating a significant overall univariate group difference *F* (1, 216) = 14.95, *p* < 0.001, *η^2^*_*p*_ = 0.07, 95% CI [0.02, 0.14], showing the control group scored significantly higher than the modified intervention group at both time points.

**Table 2:** KTK raw subtest and psychological assessments time 1 and time 2 descriptives for group and time.

	Group			
	Intervention		Control	
	T-1 (x̄ ± SD)	T-2 (x̄ ± SD)	T-1 (x̄ ± SD)	T-2 (x̄ ± SD)
WB	30.45 ± 14.19	29.17 ± 12.73	30.54 ± 13.37	32.08 ± 13.61
SH	34.56 ± 14.65	36.99 ± 15.48	37.90 ± 13.32	39.94 ± 14.01
LJ	59.12 ± 11.45	59.17 ± 11.86	58.87 ± 9.98	60.92 ± 11.26
SS	32.04 ± 7.87	33.94 ± 8.38	33.09 ± 6.37	34.53 ± 7.03
SES	14.59 ± 3.01	13.61 ± 3.32	14.00 ± 2.59	13.81 ± 3.00
SEE	17.47 ± 2.40	17.74 ± 2.58	18.74 ± 2.74*	18.89 ± 2.43*

Note. Significance is at the 0.05 level; *p < 0.05.

WB: Walking Backward, SH: Single-Leg Hop, LJ: Lateral Jump, SS: Sideways Step; SES: Self-Esteem, SEE: Perceived Physical Ability.

The two-way repeated measures MANOVA revealed a significant within-subjects main effect of time, Pillai’s Trace *V* = 0.07, *F* (6, 211) = 7.36, *p* < 0.001, *η^2^*_*p*_ = 0.17, 95% CI [0.07, 0.25], with 17.3% of the variance explained. Using the Bonferroni-adjusted criterion of *α* = .0083, significant univariate Time 1 to Time 2 were found for SH, *F* (1, 216) = 14.36, *p* < 0.001, *η^2^*_*p*_ = 0.06, 95% CI [0.02, 0.13]; SS, *F* (1, 216) = 17.76, *p* < 0.001, *η^2^*_*p*_ = 0.08, 95% CI [0.02, 0.15]; and self-esteem *F* (1, 216) = 8.28, *p* = 0.004, *η^2^*_*p*_ = 0.04, 95% CI [0.00, 0.10]. Self-esteem scores were significantly lower at Time 2 than at Time 1 across groups, whereas SH and SS scores were significantly higher at Time 2 than Time 1. WB, LJ, and Perceived Physical Ability showed no significant overall changes over time under the adjusted criterion.

In addition, one significant omnibus group x time interaction was observed, Pillai’s Trace *V* = 0.07, *F* (6, 211) = 2.52, *p* = 0.02, *η^2^*_*p*_ = 0.07, 95% CI [0.00, 0.12]. However, none of the six outcome-specific interaction tests met the Bonferroni-adjusted significance criterion of *α* = .0083. The modified intervention group decreased slightly on the WB, *F* (1, 216) = 4.94, *p* = 0.027, *η^2^*_*p*_ = 0.02, 95% CI [0.00, 0.08], while the control group improved modestly from Time 1 to Time 2. This finding was interpreted as an exploratory directional pattern only.

### 3.2. MC ability differences

Two 2 x 2 Pearson chi-square tests were conducted separately at Time 1 and Time 2. At time 1, 103 of 120 children (85.8%) in the modified intervention group and 89 of 98 children (90.8%) in the control group were classified as “Normal-to-Great.” This between group difference was not statistically significant *ꭓ^2^* = 1.28*, df* = 1*, p* = 0.30. For the second 2 x 2 chi-square test at Time 2, 106 of 120 children (88.3%) in the modified intervention group and 94 of 98 children (95.9%) in the control group were classified as “Normal-to-Great,” *ꭓ^2^* = 4.10, *df* = 1*, p* = 0.04. Although the Time 2 comparison reached the conventional significance unadjusted at *p* < 0.05 level, it did not meet the Bonferroni-adjusted significance criterion of *α* = 0.025 for the two time-specific comparisons and was therefore interpreted as a descriptive difference rather than a statistically robust between group difference. All expected cell counts exceeded 5 at both time points. These analyses explored between-group differences in Gesamt-MQ scores and their MQ classification at each assessment and were not intended to explore within-child change differences. Figs 1 and 2 display child percentages for each MQ category by group and time. This indicates that as MQ scores increased, the distribution of children across the modified intervention and control groups followed a weak but similar ordered pattern.

**Fig 1:**
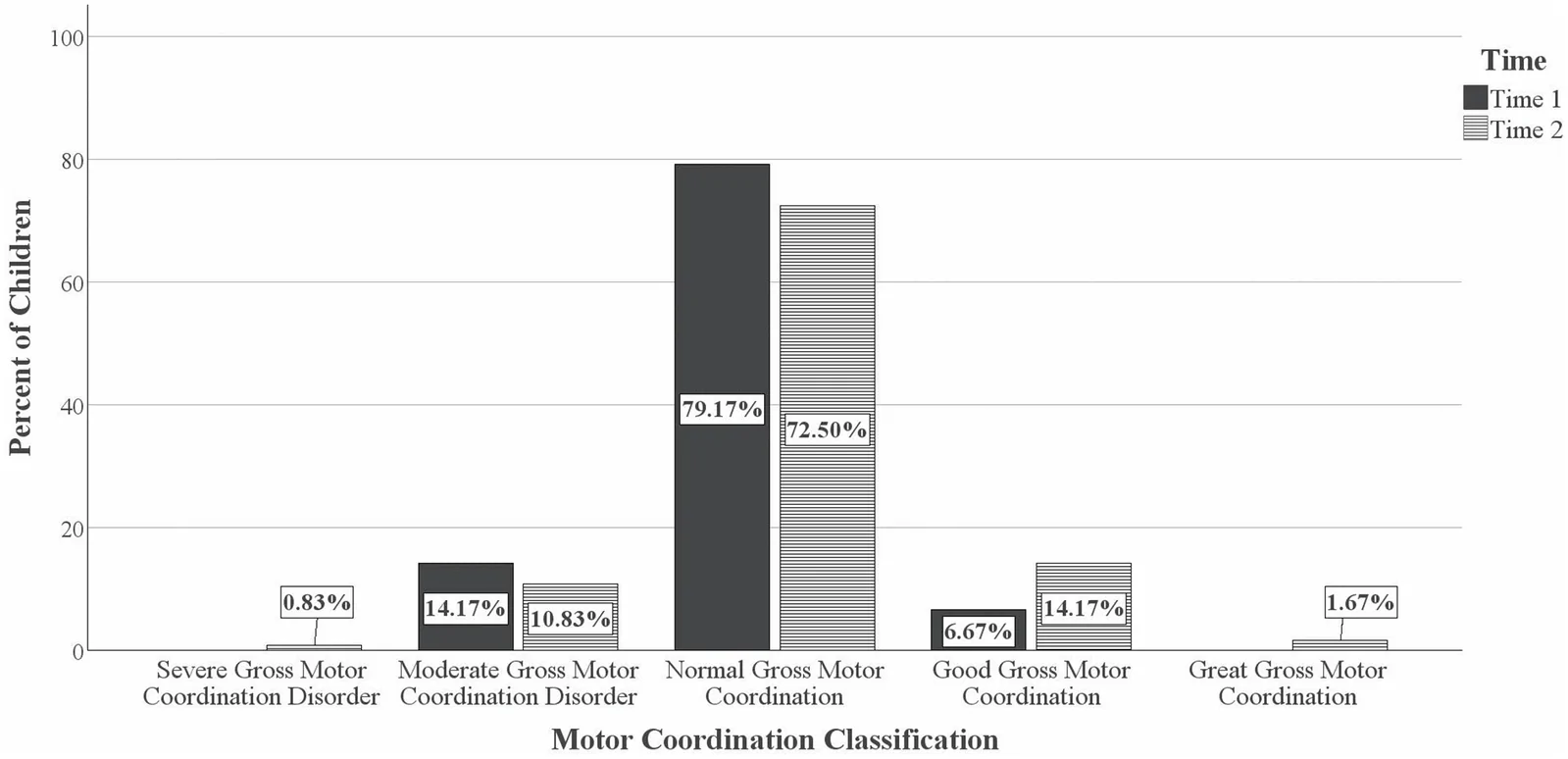
Modified intervention total motor quotient scores by time.

**Fig 2:**
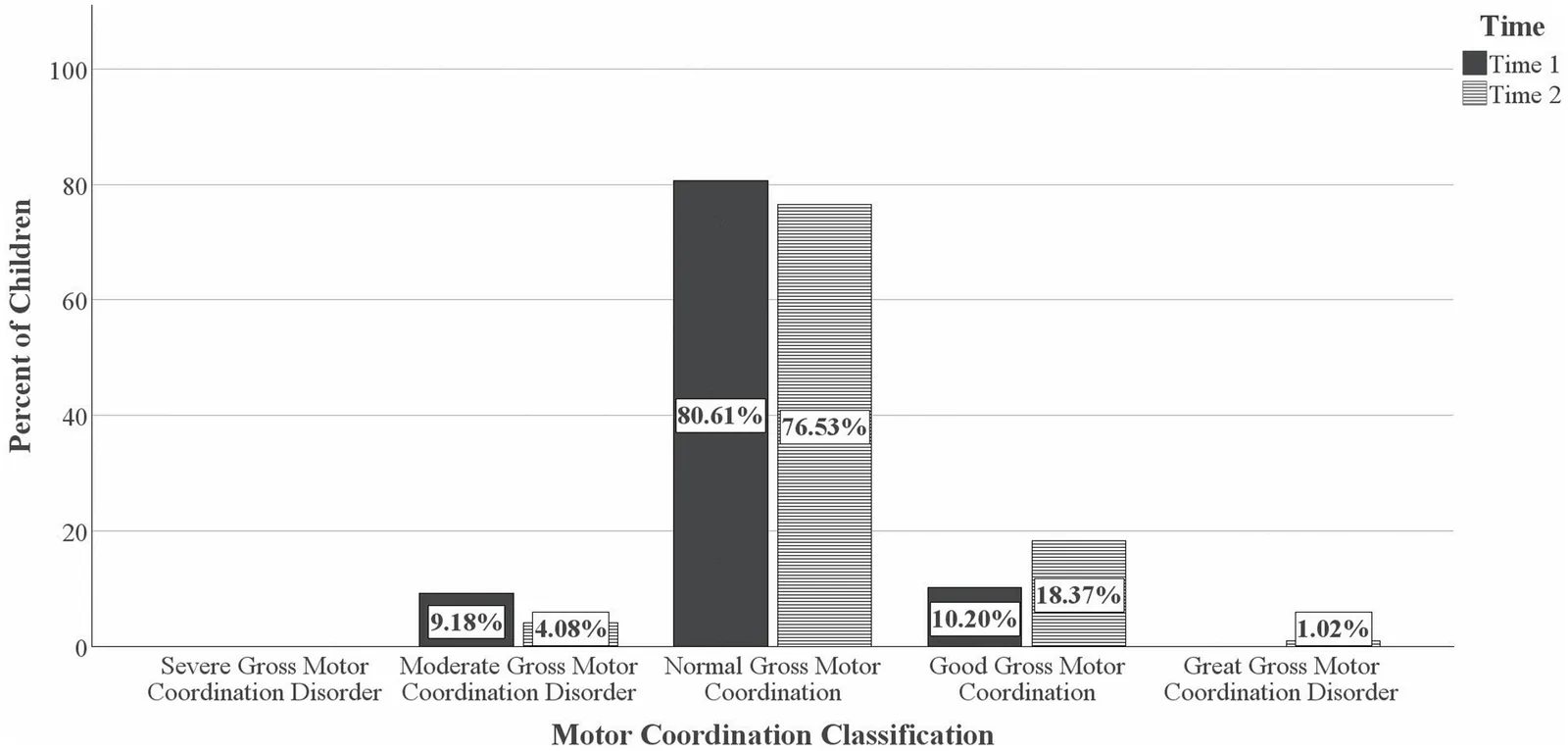
Control total motor quotient scores by time.

## 4. Discussion

The primary purpose of this exploratory study was to examine observed physiological and psychological differences and associations between fourth- and fifth- grade children who received the modified LiiNK intervention and those who did not, at two time points within one school year. The non-randomized comparative design was utilized to examine several descriptive and group differences as well as patterns of change over time.

The descriptive variables of race and body fat reflected some interesting observations. Race is somewhat challenging to match in different school districts [31,32]. This study was no different. District 1 (intervention) reflected a higher percentage of Hispanic children while District 2 reflected a higher percentage of White children. As racially different as these two school districts were, 30 minutes of recess daily seemed to reflect very similar results between the groups. Body fat was another interesting demographic for these two school districts. The bio-electrical impedance analysis (BIA) was a good assessment to determine those in healthy, overfat, and obese body fat categories based on age and sex. In the modified intervention group, approximately 55.0% to 57.5% of children were classified within the healthy body-fat category across the two assessment periods, whereas 42.5% to 45.0% were classified as overfat or obese. The control group demonstrated a comparatively larger proportion of children classified as healthy (66.3% − 69.4%) and a smaller proportion classified as overfat or obese (30.6% − 33.7%). From an observational perspective, the modified intervention group demonstrated a small shift toward the healthy classification across the study period, whereas the control group showed a small shift towards overfat or obese. This pattern is noteworthy because the modified intervention group had a different recess structure and protocols compared to the control group.

These descriptive patterns are noteworthy when compared with Farbo & Rhea (2022), who examined children receiving either 40–60 minutes or 30 minutes of daily recess and reported combined healthy/under fat classification of approximately 57–58% in the longer-recess group and 58%−61% in the 30-minute group across the school year [2]. Other studies have reported associations between excess body fat, lower motor competence, and greater constraints on participation in physical activities such as recess [3,47]. Therefore, the descriptive body-fat patterns observed in the present sample may be relevant when interpreting MC outcomes. Although group comparisons were not conducted for these descriptive variables, they are worth mentioning for future study comparisons.

### 4.1. Exploratory analyses

#### 4.1.1. Group differences.

This study focused on three key variables, MC, PPASC, and SES with fourth and fifth grade children from two different school districts, both receiving 30 minutes of recess but different protocols. A key exploratory trend observed was the control group scored significantly higher on the PPASC compared to the modified LiiNK intervention group, despite both groups receiving a similar recess duration. Due to the control group having higher scores at Time 1 and remained higher at Time 2, this pattern showed a pre-existing group difference rather than a change associated with the modified recess condition. The control group’s recess included transition time, effectively shortening the active play period to less than 30 minutes, which may have led to intense, concentrated bouts of activity that bolstered perceptions of ability. Another potential explanation is that the children in the modified intervention group had previously experienced the full 60-minute model, therefore, the reduction to 30 minutes may have led to decreased opportunities for consistent motor skill practice and physical engagement. This reduction could have been perceived negatively, possibly diminishing their confidence and satisfaction with their physical capabilities. This possibility aligns with Farhat et al. [18], who found that children’s perceived physical abilities are closely tied to opportunities for motor skill mastery. Since the groups were not randomly assigned and the analyses did not adjust for school-, district-, or demographic differences, the observed PPASC difference cannot be attributed specifically to recess duration and protocols. Therefore, it is best to interpret these findings as an exploratory between-group observation.

Further, the total group reflected MC subtest improvements for SH and SS. SH primarily reflects vertical strength, rhythm, and balance [16]. Because both tasks require repeated coordinated movement, the observed gains may reflect developmental progression and continued exposure to unstructured outdoor play across the school year rather than an effect of one recess condition. Moreover, these specific MC patterns utilized at recess are commonly practiced during unstructured recess through hopping, jumping, stepping, and other coordinated locomotor activities [11,17,21,49]. Prior literature further suggests that repeated play-based movement experiences and physical activity participation can support the development of motor competence by providing frequent opportunities to practice rhythm, balance, strength, and coordination [14,21,30,50]. Because improvements in SH and SS were observed across both recess conditions, the present findings are more consistent with movement experiences shared by both groups than with an effect unique to the modified LiiNK protocol. Campbell-Pierre et al, reported more favorable MC outcomes among children participating in the full 60-minute LiiNK intervention than children receiving shorter recess durations. Together, these findings suggest that repeated motor-skill practice may contribute to specific MC gains, while the amount and structure of recess exposure remain important factors for future investigation.

In contrast, the observed total group significant decrease in self-esteem from Time 1 to Time 2 must be interpreted with considerable caution due to the marginal reliability of the scale. Additionally, discrepancies in the literature have been found about this age group and their self-esteem levels. Fourth and fifth grade children have demonstrated lower self-esteem at this age level as a result of changes in emotional and social insecurities [26]. Other studies have shown if MC and physical self-concept are better, then self-esteem will be better [20,41]. Since the total group was similar, not only can 30 minutes of recess daily not be tied to lower or higher self-esteem, but the self-esteem scale may not have been the best scale to use for this age group. In order to know if recess with this age group can influence MC, perceived physical ability, and self-esteem better, a study examining the full LiiNK intervention with the modified version are needed to clarify the impact of recess duration. The four-month interval between time points may also have been insufficient in detecting meaningful changes in self-esteem for the total group or a comparison of the two groups.

Although the overall multivariate group × time interaction was significant, none of the individual outcome-specific interactions remained significant after adjusting for multiple comparisons; therefore, no specific physiological or psychological outcome was interpreted as demonstrating a reliable differential pattern between groups over time.

#### 4.1.2. MC ability differences.

The absence of significant total MQ score group differences in our analysis represents an observed trend worth noting. The Gesamt-MQ classification findings provide a descriptive MC proficiency picture within the two recess conditions. Since the exploratory Gesamt-MQ classification findings indicated that the majority of children in both groups were classified within the “Normal-to-Great” MQ range, this suggests that a 30-minute recess duration may be able to differentiate MC scores or potential disorder classifications from the KTK in elementary-aged children. However, this finding should be interpreted with caution as it contrasts with Campbell-Pierre et al., [16], where the children in the full 60-minute LiiNK recess intervention were classified at 85% in the “Normal-to-Great” MQ range than the control group with 30 recess minutes daily at 79%. The present findings should not be interpreted as evidence that 30 minutes of recess is sufficient to produce or maintain a particular level of MC; rather, they describe the distribution of Gesamt-MQ classifications observed in this sample under two different 30-minute recess conditions.

KTK MQ classifications are able to distinguish children who have severe gross motor coordination disorders to great gross motor coordination, with the four subtests assessing vertical balance, rhythm, postural control, and lateral agility. In this particular sample, the majority of both groups scored in the “Normal-to-Great” MC range in Time 1 and Time 2. Based on this observation, detecting MC issues through MQ classifications on the KTK still holds clinical and educational value, allowing schools and professionals to allocate resources for early intervention through physical education classes. Classification changes across time should be interpreted cautiously because temporary physical restrictions, such as braces, casts, or medically limited use of a limb, may influence performance on individual KTK tasks.

Consequently, the observed differences between previous studies and the present study may suggest that while 30-minutes of recess supports observation and screening for motor delays, it may not be adequate to produce the same developmental gains seen with longer recess durations. Overall, these results provide no evidence that the modified intervention benefits the children any better than having 30 minutes of recess without the modified protocols.

### 4.2. Limitations and future research

This study has several limitations that may affect the generalizability and interpretation of the findings. The use of convenience sampling introduced selection bias and reduced the applicability of the findings to broader educational or demographic contexts. Consequently, we cannot rule out the possibility that observed group differences reflect school-level characteristics or clustering effects rather than the recess protocol themselves. Furthermore, children were nested within schools and districts, and clustering was not incorporated into the primary statistical models or the original power analysis. The absence of fidelity measures, such as actual outdoor time, physical activity intensity, weather related-cancellations, outdoor space, and attendance during recess, limits the ability to confirm that the modified intervention was implemented consistently across the study period.

Several limitations also affected interpretation of the changes observed over time. The four-month assessment period included a winter break and may have been too short to fully capture developmental changes in MC or psychological outcomes. The study also assumed that all children had sufficient cognitive capacity to understand instructions, respond accurately to questionnaires, maintain motivation, and exert full effort during physiological assessments, which may not account for individual variability in comprehension or motivation. The demographic differences between groups, particularly in racial composition and body fat classification, represent potential confoundedness that were not statistically adjusted for in our exploratory analyses. The psychological measures used to assess self-esteem and perceived physical ability were subjective and self-reported, meaning there was room for individual interpretation. The marginal reliability of the self-esteem measure (Cronbach’s α = 0.66) also warrants caution in interpreting findings related to this construct.

MQ classification analyses also had limitations. Gesamt-MQ classifications were used to discretely distinguish children as “Below-Normal” or “Normal-to-Great,” but temporary physical conditions such as braces or restricted use of a limb were not systematically documented and could have influenced performance on individual KTK tasks. Additionally, preliminary assumption screening identified modest departures from univariate normality for several outcomes, while Box’s M test suggested heterogeneity of covariance matrices across groups. Pillai’s Trace was used because of its relative robustness to departures from these assumptions. These findings do not devalue the analysis but reinforce the need to interpret the multivariate results cautiously and in conjunction with the observed effect sizes and descriptive patterns.

Future research should focus on understanding the long-term effects that longitudinal studies can offer regarding the implementation of recess interventions such as LiiNK. Longer time between data collection periods with additional longitudinal data points would help capture changes over time and assess the sustainability of physiological and psychological outcomes. Extending the follow-up period through the end of the school year, with the second assessment occurring in May rather than February, may also provide a more complete evaluation of developmental change and the potential effects of sustained recess exposure. Exploring diverse populations could provide further insights into how cultural, socioeconomic, and environmental factors influence the effectiveness of recess interventions. By incorporating more demographically balanced and randomized samples to future studies would improve generalizability and reduce potential selection bias. Moreover, using both objective and subjective assessment tools would provide a more intuitive understanding of children’s holistic development.

Standardizing measurement tools and ensuring age verification procedures in future studies will also further enhance methodological rigor. Finally, future research should examine MC and perceived physical ability changes when information can be gathered on types of equipment, loose parts, descriptions of space available, and time given for independent play for a better understanding of MVPA, types of movement, and obesity prevalence. Previous research has shown these facets of play environments could interfere with children’s MC performance and self-perceptions of physical ability [16,17,19,50]. Direct comparison of the full 60-minute LiiNK intervention, the modified 30-minute model, and a 30-minute control condition would provide a stronger examination of whether recess duration or associated protocols are related to MC development.

### 4.3. Practical implications

While this exploratory study does not establish causal effects; the observed patterns suggest several practical considerations for schools and educators that permits further deliberation. Rather than supporting a specific recess schedule, the findings reinforce the importance of examining recess duration, frequency, play environment, and implementation together when considering children’s physiological and psychological development. Schools may consider increasing the number of recess opportunities that are offered outdoors and unstructured by implementing the full LiiNK intervention. Studies have shown that providing four 15-minute daily recess breaks in elementary schools is associated with significantly better motor skill development, PA, social interaction and psychological well-being than those with 30-minutes or less of recess daily [15,51].

The exploratory demographic findings highlight the importance of considering individual and population characteristics when interpreting school-based physiological and psychological outcomes. Differences in age or grade level, biological sex, racial composition, and body-fat classifications may contribute to variation in motor coordination, physical activity participation, and related developmental outcomes [3,39,47]. Although these variables were descriptive and were not statistically adjusted for the present study, school personnel and researchers should consider demographics when comparing groups, interpreting screening results, and designing future recess or physical activity programs. In addition, schools should consider implementing routine MC screenings in PE settings, utilizing the KTK. These screenings can assist in identifying children with MC deficits who may benefit from additional assessment or support. The present study’s purpose was not to establish that MC difficulties are attributable to reduced recess. Rather, it demonstrates that expanding the number of recess periods provided in schools daily could help children be less sedentary, more physically active, and feel better about who they are socially and emotionally.

The psychological and MQ classification findings also have practical implications for schools. Although self-esteem scores were lower at Time 2 across both groups, this pattern was not specific to either recess condition, suggesting that psychological outcomes should be considered alongside physical outcomes when evaluating recess programming. Schools may benefit from monitoring self-esteem and perceived physical ability in addition to MC, particularly during fourth and fifth grade when developmental and social changes may influence children’s self-perceptions. Similarly, the high proportion of children classified within the “Normal-to-Great” MC range at both time points demonstrates the potential value of the KTK to assess MC proficiency within school populations. However, because neither time-specific chi-square comparison met the Bonferroni-adjusted significance criterion, these percentages should not be interpreted as evidence that either 30-minute recess condition produced superior MC outcomes. Rather, they support the use of continued MC screening to identify children who fall below expected levels and may benefit from additional movement opportunities or support.

By integrating PA and play within the broader spectrum of the school curriculum, it may further support developmental benefits. Unstructured outdoor play has been consistently identified as a crucial context for fostering self-awareness, emotional regulation, and physical confidence [51,52]. These periods of providing a physically active environment are instrumental in enhancing emotional intelligence and perceived competence, both of which are vital for success in social interactions and personal physical growth. By equipping PE teachers and child development professionals with data from screenings, schools can make informed decisions about which students may require MC intervention, ensuring that reduced recess time does not further disadvantage children already at risk.

### 4.4. Conclusion

This exploratory study offers preliminary insight into the complex role of recess (unstructured outdoor play) through all stages of child development, suggesting that its benefits extend beyond simple duration. While both groups increased in selected MC outcomes, self-esteem decreased modestly over time across both groups. The exploratory WB pattern, characterized by a slight decline in the modified intervention group and a modest improvement in the control group, suggests that balance and postural-control outcomes warrant further investigation; however, the small effect and lack of significance after Bonferroni adjustment preclude definitive conclusions. The control group reported higher perceived physical ability at both times. This difference was already present at Time 1, therefore, it may represent a pre-existing between-group difference rather than an effect of recess structure. However, these interpretations remain speculative given the non-randomized, comparative exploratory design and should be tested in future research.

Ultimately, this exploratory study did not identify a consistent pattern of more favorable physiological or psychological outcomes for the modified 30-minute LiiNK condition compared with the 30-minute control condition. These findings should not be interpreted as evidence regarding the effectiveness of the full 60-minute LiiNK model, which was not directly evaluated in the present study. Direct comparison of the full 60-minute LiiNK intervention, the modified 30-minute model, and a 30-minute control condition is needed to better distinguish the potential roles of recess duration, frequency, and associated protocols. Future research using larger and more diverse samples, longer follow-up periods, fidelity measures, adjustment for baseline and demographic differences, and statistical approaches accounting for school-level clustering is warranted to clarify these exploratory findings.

## Supporting information

S1 File Data Information. (XLSX)
